# The roles of immune factors in neurodevelopment

**DOI:** 10.3389/fncel.2025.1451889

**Published:** 2025-04-10

**Authors:** Chong Wang, Tingting He, Jie Qin, Jianwei Jiao, Fen Ji

**Affiliations:** ^1^State Key Laboratory of Organ Regeneration and Reconstruction, Institute of Zoology, Chinese Academy of Sciences, Beijing, China; ^2^School of Life Sciences, University of Science and Technology of China, Hefei, China; ^3^University of Chinese Academy of Sciences, Beijing, China; ^4^Beijing Institute for Stem Cell and Regenerative Medicine, Beijing, China

**Keywords:** cytokine, microglia, meninges, maternal immune activation, immunity

## Abstract

The development of the nervous system is a highly complex process orchestrated by a multitude of factors, including various immune elements. These immune components play a dual role, not only regulating the immune response but also actively influencing brain development under both physiological and pathological conditions. The brain’s immune barrier includes microglia in the brain parenchyma, which act as resident macrophages, astrocytes that support neuronal function and contribute to the inflammatory response, as well as circulating immune cells that reside at the brain’s borders, including the choroid plexus, meninges, and perivascular spaces. Cytokines—soluble signaling molecules released by immune cells—play a crucial role in mediating communication between immune cells and the developing nervous system. Cytokines regulate processes such as neurogenesis, synaptic pruning, and inflammation, helping to shape the neural environment. Dysregulation of these immune cells, astrocytes, or cytokine signaling can lead to alterations in neurodevelopment, potentially contributing to neurodevelopmental abnormalities. This article reviews the central role of microglia, astrocytes, cytokines, and other immune factors in neurodevelopment, and explores how neuroinflammation can lead to the onset of neurodevelopmental disorders, shedding new light on their pathogenesis.

## Introduction

For a long time, the central nervous system (CNS) was considered an immune-privileged organ due to protective mechanisms such as the blood-brain barrier (BBB). However, emerging evidence indicates that the brain maintains active interactions with the peripheral immune system through meningeal lymphatic vessels, with leakage occurring in specific regions of the BBB (Rustenhoven et al., 2021). These breaches allow immune cells to infiltrate, initiating a cascade of immune responses that influence CNS function.

Neurons, being non-renewable, reach a stable state upon maturation but are progressively damaged and lost with aging. To preserve neuronal integrity and function during this process, microglia, the primary immune cells of the CNS, play an essential role by mediating inflammatory and immune responses, mitigating nervous system disorders, and maintaining brain homeostasis (Ransohoff and Cardona, 2010). Beyond inflammation, the interaction between neurons and bone marrow-derived cells significantly impacts CNS health. Disruptions in this interaction, such as microglial loss, impair synaptic construction and weaken neuronal communication, leading to deficits in information processing and transmission. Such dysfunctions are linked to social deficits and mental disorders, including autism (Parkhurst et al., 2013; Zhan et al., 2014).

The immune system’s role extends beyond pathogen defense, as it is critical for the proper development and maintenance of the nervous system (Becher et al., 2017; Deverman and Patterson, 2009). Immune cells and their associated factors are indispensable for neural processes such as growth, differentiation, migration, synapse formation, and the preservation of myelin sheaths (Ferro et al., 2021). Physiological functions of the nervous system are, therefore, intricately tied to immune system dynamics. For instance, microglia not only maintain tissue homeostasis and mediate injury responses but also participate in synaptic pruning, cellular debris clearance, and apoptotic cell elimination—processes critical for optimizing neuronal and glial function (Cowan and Petri, 2018; Ferro et al., 2021).

Furthermore, border-associated macrophages (BAMs), found in the meninges, perivascular spaces, and ependymal regions, contribute to CNS immunity. The meninges, in particular, host a diverse array of immune cells, which act as significant sources of cytokines. Immune dysregulation has been strongly associated with the onset of neuropsychiatric disorders, many of which may originate during fetal development. For example, maternal infections during pregnancy can disrupt both maternal and fetal immune systems, interfering with fetal brain maturation and predisposing offspring to conditions such as autism, schizophrenia, and other neurological disorders (Han et al., 2021; Solek et al., 2018).

This emerging understanding of the interplay between the immune and nervous systems highlights their mutual influence in both health and disease. Neuroinflammation, while protective, contributes to neurodegenerative diseases like Parkinson’s and Alzheimer’s. Therapeutic strategies such as stem cell therapy, genetic interventions, and nanoparticles show promise in modulating inflammation and slowing disease progression, revealing new opportunities for therapeutic interventions targeting immune-mediated neurological disorders (Adamu et al., 2024). In this article, we categorized the immune factors that contribute to brain development into several broad categories: cytokines, microglia, astrocytes, and the diverse immune cells found at the brain border. Their role in normal neurodevelopment and how they relate to specific neurodevelopmental disorders are discussed.

### Physiological role of cytokines in normal brain development

Cytokines are a diverse family of proteins including chemokines, interferons (IFNs), interleukins (ILs), tumor necrosis factors (TNFs), and other sub-families, which perform a wide array of physiological functions (Table 1) (Ampie and McGavern, 2022). Their roles are multifaceted and context-dependent. For instance, IL-1β enhances neuronal excitability by increasing Ca^2+^ flux through NMDARs during glutamatergic synaptic transmission. Conversely, IL-1β can inhibit neuronal excitability by reducing the ion flux through voltage-gated Na^+^ (Nav) (Matcovitch-Natan et al., 2016), Ca^2+^ (Cav), and K^+^ (Kv) channels (Vezzani and Viviani, 2015; Zipp et al., 2023). This review focuses on the roles of cytokines during normal central nervous system (CNS) development.

**Table 1:** Cytokines listed and their functions in brain development.

Cytokine	Function in brain development	References
IL-1β	Changes neuronal activity and synaptic transmission; activates microglia, and enhances immune cell recruitment	Hewett et al. (2012)
CNTF	Maintains RGC populations and promoting their differentiation into astrocytes	Jones and Jenkins (2018), Kotasová et al. (2014), Kummer et al. (2021), and Nicola and Babon (2015)
IL-6	Regulates the proliferation and differentiation of neural stem cells	Erta et al. (2012), Hunter and Jones (2015), and Rothaug et al. (2016)
CXCL12	Regulates the generation and migration of newborn granule neurons	Watson et al. (2020)
CXCL1, CXCL8	Regulates gliogenesis	Tsai et al. (2002), Turbic et al. (2011), and Wang et al. (2008)
C1, C3, and C4	Regulates growth and pruning of synapses	Coulthard et al. (2017), Sekar et al. (2016), and Stevens et al. (2007)
TGFβ	Regulates remyelination	Miron et al. (2013)
TNF-α	Regulates processes such as neuronal cell proliferation, differentiation, apoptosis, and synaptic pruning	Linnerbauer et al. (2020) and Liu et al. (2017)
IL-17	Increases the frequency of excitatory postsynaptic currents	Giovannoni and Quintana (2020) and Linnerbauer et al. (2020)
IL-6	Enhances glutamatergic synapse formation and functional connectivity; selectively promotes synaptic formation	Ng et al. (2018) and Patel et al. (2019)
IFN-γ	Exert diverse effects on the neuronal survival, proliferation, differentiation, synapse formation, and migration	Becher et al. (2017)

Cytokines are not only essential for immune system function but also play important roles in normal CNS development. During brain development, cytokines regulate neurogenesis, support neuronal survival, and influence synaptic pruning. They are also involved in modulating synaptic transmission and synaptic plasticity (Deverman and Patterson, 2009). Cytokines and their receptors are widely distributed across neurons, astrocytes, microglia, and oligodendrocytes, underscoring their functional diversity. Dysregulation of cytokines signaling can result in developmental abnormalities (Ampie and McGavern, 2022; Cowan and Petri, 2018).

Cytokines such as leukemia inhibitory factor (Louveau et al., 2018), ciliary neurotrophic factor (CNTF), and IL-6 signal through GP130 receptor to regulate CNS development (Jones and Jenkins, 2018; Kotasová et al., 2014; Kummer et al., 2021; Nicola and Babon, 2015). GP130 activation, which predominantly signals via STAT3, is essential for maintaining radial glial cell (RGC) populations and promoting their differentiation into astrocytes (Nakashima et al., 1999). In GP130-deficient mice, RGC marker expression and mitotic activity are reduced, while STAT3 knockdown accelerates cell differentiation (Kotasová et al., 2014). Moreover, GP130-STAT3 signaling enhances GFAP expression, with astrocyte populations significantly diminished in GP130-knockout models (Barnabé-Heider et al., 2005; Bonaguidi et al., 2005; Deverman and Patterson, 2009). Trophic factors, including brain-derived neurotrophic factor (BDNF), and glial cell line-derived neurotrophic factor (GDNF), are vital for neuronal survival, synaptic plasticity, and glial cell function (Costa et al., 2022; Wright et al., 2021). Although they play a crucial role in neural homeostasis and modulating inflammatory responses, they are less readily measurable in biofluids like cerebrospinal fluid (CSF) or blood compared to cytokines. Additionally, their therapeutic application is hindered by the challenge of crossing the BBB, often requiring specialized delivery systems such as viral vectors or nanoparticles, which limits their immediate use in diagnostics and treatment (Lindholm and Saarma, 2022; Zhou et al., 2020).

IL-6 is a versatile cytokine with critical functions in both physiological and pathological contexts (Erta et al., 2012; Hunter and Jones, 2015; Rothaug et al., 2016). It regulates the proliferation and differentiation of neural stem cells, acting as a neurotropic factor during neurogenesis (Bowen et al., 2011). IL-6 overexpression promotes forebrain neural stem cell proliferation in postnatal mice, while IL-6 receptor (IL-6R) knockout results in a long-term reduction of these stem cells (Kathuria et al., 2022). However, excessive IL-6 expression can exacerbate neurological disorders such as autism, schizophrenia, and Alzheimer’s disease by promoting immune-driven inflammation (Ng et al., 2018; Upthegrove and Khandaker, 2020; Wei et al., 2013). The exact cause of this phenomenon remains to be explored. Recent research showed that injecting IL-6 into pregnant mice or embryos promoted synaptogenesis in the offspring. This process directly affects excitatory neurons through the IL-6R, leading to an increased number of glutamatergic synapses (Mirabella et al., 2021). Unlike the inflammatory effects commonly associated with IL-6, this synaptogenic process specifically targets neurons, emphasizing a distinct, non-inflammatory mechanism. The observed increase in excitatory synapses, without a corresponding rise in inhibitory synapses, indicates an excitatory/inhibitory imbalance. Such an imbalance is a recognized pathological hallmark of neurodevelopmental disorders, including autism spectrum disorder and schizophrenia (Nelson and Valakh, 2015; Sohal and Rubenstein, 2019). The expression of IL-6R is stable in neurons during early developmental stages but diminishes in later phases, suggesting a temporal specificity in its function. This reduction in IL-6R expression in mature neurons implies that IL-6-mediated signaling predominantly impacts early neural development (Campbell et al., 2014; Mirabella et al., 2021). Differences between IL-6R and soluble (s) IL-6R have been extensively reviewed (Mihara et al., 2012). During early development, classical IL-6 signaling is the dominant mechanism. However, studies in the mature brain indicate that IL-6 also can exert pathogenic effects through trans-signaling (Willis et al., 2020).

IL-1β, another cytokine, plays an important role in brain development and function. It is especially crucial in neuronal development, synaptic transmission, microglial activation, and nervous system repairment. IL-1β exerts its effects by influencing CNS glial cells to regulate inflammatory responses, partly through the activation of the nuclear factor-κB (NF-κB) transcription factor. This activation triggers a cascade of events, inducing the expression of other cytokines that exert specific effects on neurons and glial cells of the CNS via a variety of signaling pathways (Hewett et al., 2012). The intricate interplay of IL-6 and IL-1β in neural development highlights the complex roles of cytokines in the CNS. While these molecules are essential for normal brain development, their dysregulation can contribute to the etiology of neurodevelopmental disorders, warranting further investigation into their underlying mechanisms and broader implications.

Under normal physiological conditions, chemokines and their receptors are extensively expressed in the nervous system, including neurons, endothelial cells, and oligodendrocytes. These molecules play a critical role in mediating cell-to-cell communication and ensuring the proper functioning of the nervous system (Callewaere et al., 2007; Zhang et al., 2017). For instance, the C-X-C motif chemokine 12 (CXCL12), also referred to as stromal cell-derived factor 1 (SDF-1), regulates the generation and migration of newborn granule neurons in the SGZ by binding to its CXCR4 receptor (Abe et al., 2018; Bhattacharyya et al., 2008). Furthermore, CXCL12 facilitates the maturation and differentiation of oligodendrocyte precursor cells and mediates the interaction between microglia and neural stem cells (Watson et al., 2020). Other chemokines such as CXCL1, CXCL8, and CCL21 are similarly essential for gliogenesis (Tsai et al., 2002; Turbic et al., 2011; Wang et al., 2008). In addition to their physiological roles, chemokines have been extensively implicated in several neurodevelopmental disorders, including multiple sclerosis (Cui et al., 2020), autism spectrum disorder (ASD) (Ye et al., 2021), and bipolar disorder (Misiak et al., 2020). For example, elevated levels of chemokines such as MCP-1 and RANTES have been detected in the brains and CSF of individuals with ASD. In certain children with ASD, the relationship between the chemokines and the observed behavioral impairments could hold diagnostic relevance in terms of the disorder’s etiology. Similarly, individuals with bipolar disorder and multiple sclerosis frequently exhibit elevated concentrations of specific chemokines, underscoring their relevance in these pathologies (Ashwood et al., 2011; Brietzke et al., 2009).

Research into the functions of complement in neurodevelopment primarily focused on its involvement in pre- and postnatal synaptic pruning (Coulthard et al., 2017; Südhof, 2018). Key complement components, including C1, C3, and C4, are critical for the growth and pruning of synapses, thereby shaping neural circuits (Coulthard et al., 2017; Sekar et al., 2016; Stevens et al., 2007). Beyond synaptic pruning, the complement system has been implicated in the broader regulation of neural development. Similar to other immune molecules, complement proteins are synthesized and secreted by various cell types within the brain and serve diverse functions during CNS development, such as supporting neurogenesis, neuronal differentiation, and synaptic refinement (Tenner et al., 2018). Under normal conditions, complement receptors, such as C3aR and C5aR, are expressed in neural stem cells to regulate their development (Gorelik et al., 2017; Magdalon et al., 2020). For instance, C3aR has been shown to inhibit neural stem cell proliferation. Mouse embryos treated with C3aR antagonists exhibit increased NPC proliferation in the ventricles or subventricular zones. C3aR can also regulate the differentiation of stem cells *in vitro* and affect cell migration by interacting with CXCL12 (Coulthard et al., 2018). Similarly, C5aR1 plays a crucial role in promoting the proliferation and differentiation of neural progenitor cells through a signaling cascade involving protein kinase C Zeta. Disruption of C5aR1 signaling during neurogenesis can result in adverse outcomes for brain development, leading to the occurrence of behavioral abnormalities (Coulthard et al., 2017).

Cytokines can be consistently measured in CSF, blood, and tissue across species, making them valuable biomarkers for neuroinflammatory diseases (Tsumura et al., 2022). Certain cytokines can cross the BBB or alter its permeability, directly impacting immune responses in the CNS (Yang et al., 2022). This ability supports innovative neuroimmune regulation strategies, such as cytokine-neutralizing antibodies (e.g., anti-TNF-α therapies) and cytokine receptor antagonists, broadening their potential in disease treatment (Jesudasan et al., 2021; Reale et al., 2021). In short, CNS injuries, infections, and psychological stressors can induce cerebral cytokine production, resulting in significant alterations in brain function and behavior, it is necessary for further investigation into the role of cytokines in neuroinflammation and their modulation of neural activity (Konsman, 2022).

### Immunity and development: from the brain parenchyma to the brain border

Adult neurons exhibit limited regenerative capacity, resulting in the production of only a small number of new neurons in the adult brain. Consequently, it is critical to protect the neurons from pathogens and maintain a normal physiological environment for optimal brain function. The brain possesses a distinct immune landscape characterized by unique features. Under physiological conditions, microglia serve as the primary immune cells in the brain. However, in the pathological state, peripheral lymphocytes are capable of infiltrating the brain to assist in pathogen elimination. In contrast to the brain parenchyma, the meninges harbor a substantial population of immune cells, and the intensity and nature of the “boundary” immune response in these regions can profoundly influence the brain’s physiological state.

### Microglia

Microglia, the brain-resident immune cells, constitute approximately 10% of all CNS cells (Salter and Stevens, 2017). These cells play indispensable roles in both physiological and pathological conditions. Derived from the yolk sac, microglia populate the CNS during early embryonic development (Ginhoux et al., 2010). As immune sentinels, microglia perform a wide range of critical functions, including mitigating pathogenic infections, preventing nerve damage, clearing pathogens and damaged cells, participating in synaptic remodeling, and regulating neuronal proliferation, differentiation, and apoptosis. Through these diverse activities, microglia are essential for maintaining CNS homeostasis (Colonna and Butovsky, 2017; Hickman et al., 2018).

Microglia represent a highly heterogeneous cell population, with significant phenotypic diversity revealed through advancements in sequencing technologies (Masuda et al., 2020). This heterogeneity is particularly pronounced during early development and appears to be conserved across species (Geirsdottir et al., 2019; Masuda et al., 2020). In both mice and humans, microglial heterogeneity is greatest in the early developmental stages and decreases as development progresses (Kracht et al., 2020; Matcovitch-Natan et al., 2016). Single-cell sequencing analyses of developing microglia revealed that microglia migrated from the yolk sac to the brain in an immature state and these cells had highly heterogeneous gene expression profiles. While these immature microglia lack immune-related functions during early stages, they begin to acquire immune capacities after embryonic after E14.5. The acquisition is accompanied by a reduction in microglial heterogeneity, leading to a more uniform population in adulthood (Matcovitch-Natan et al., 2016). Human studies have produced similar findings. Analysis of microglial single-cell gene expression profiles from human fetuses at gestational weeks 9–18 has demonstrated that microglia in humans are heterogeneous during the early stages of development as well. These microglial gene networks are associated with cell cycle regulation (E2F2), morphogenesis (SOX4/SOX11), and chromatin remodeling (SP1) (Kracht et al., 2020). Importantly, gene expression patterns vary across brain regions, such as the cerebral cortex, diencephalon, midbrain, and cerebellum. As in mice, microglial heterogeneity in the human decreases as these cells acquire immune-related functions culminating in a more homogenous population in adulthood, primarily focused on immune surveillance. The inherent heterogeneity of microglia has prompted the development of a conceptual framework and recommendations for standardized microglial nomenclature (Paolicelli et al., 2022). Microglial diversity is driven by signals from the CNS microenvironment, which induce dynamic transcriptional changes throughout development. Additionally, factors such as age, spatial location, nutritional status, and microbiome composition significantly influence microglial properties and functions. These dynamic influences make it challenging to precisely define microglial states, their transitions, or their numbers. Consequently, a universal framework for defining microglial states and developing standardized nomenclature is essential for advancing the field.

**Figure 1:**
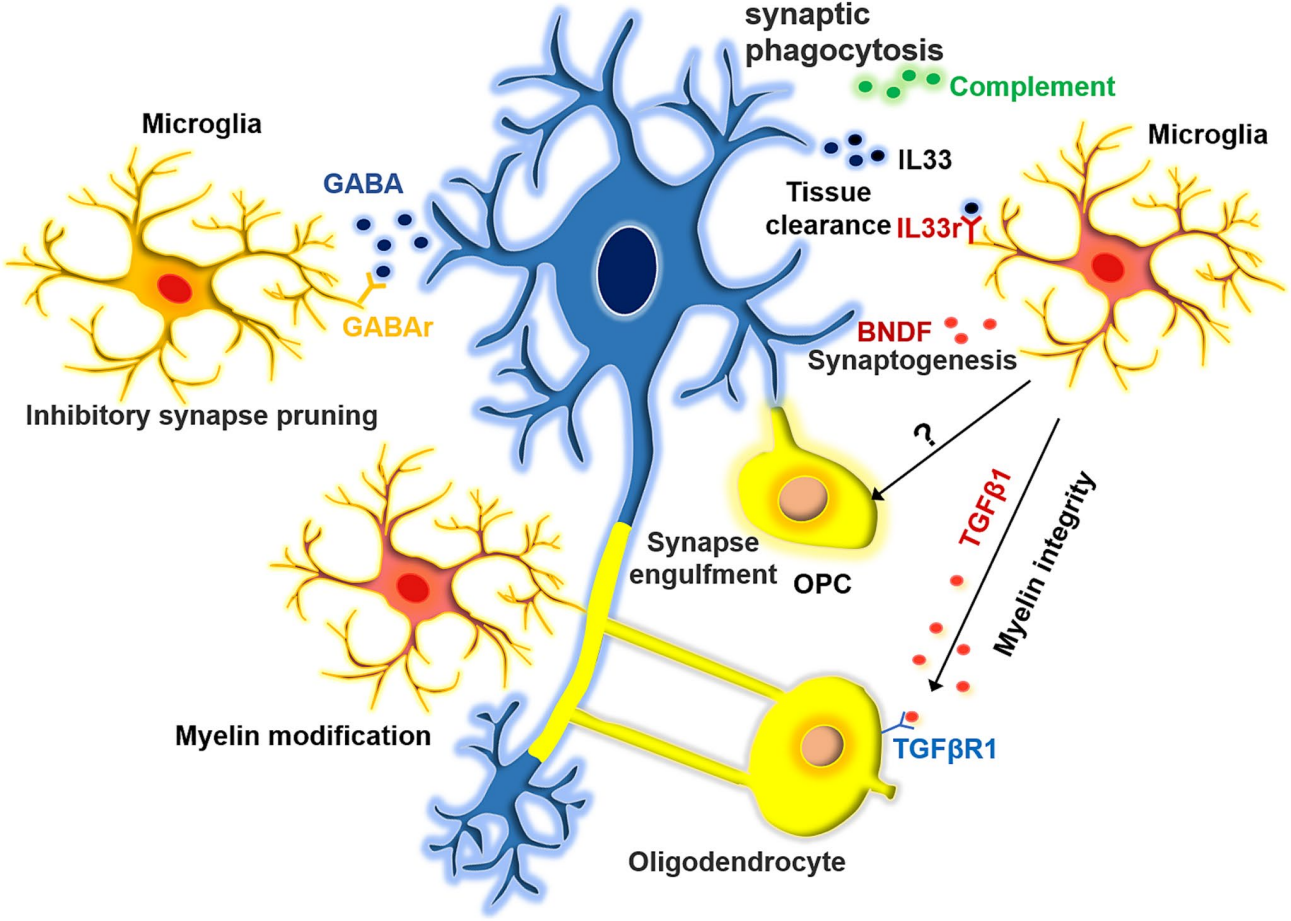
The function of microglia in synapse pruning and myelin modification. Microglia exhibit a range of effects on synapses, displaying selectivity in their influence on both excitatory and inhibitory synaptic pruning. Moreover, they facilitate synaptic formation by the extracellular matrix. Additionally, microglia play a role in orchestrating the development of myelin sheaths around axons. They can modulate the structural integrity of these sheaths either by influencing oligodendrocytes indirectly or by directly engulfing them.

The high degree of microglial heterogeneity contributes to functional complexity (Figure 1). One of the key functions of microglia is synaptic pruning. TREM2 is an innate immune receptor on microglia that is essential for synaptic refinement during the early stages of brain development. TREM2 depletion impairs synapse elimination, which ultimately increases excitatory neurotransmission and reduces long-range functional connectivity (Filipello et al., 2018). In general, microglia-mediated synaptic phagocytosis depends on the complement system (Matsuda, 2017). However, microglial subsets may play different roles in this process. In addition to the pruning of excitatory synapses, certain microglial subsets can trim inhibitory synapses. Microglia expressing GABA receptors are capable of selectively engulfing inhibitory GABA synapses. Mice lacking GABA-receptor-expressing microglia have more inhibitory synaptic connections and show higher levels of inhibitory neuronal signaling, despite the activity of their excitatory synapses remaining unaffected (Favuzzi et al., 2021). Besides pruning synapses, microglia promote synaptic growth and learning-induced synapse formation by secreting BNDF and directly interacting with neurons (Parkhurst et al., 2013). Microglia-derived BDNF regulates neuronal development in the medial prefrontal cortex and is associated with abnormal behavior. Transgenic mice overexpressing microglia BDNF exhibit impaired sociability and excessive mPFC inhibitory neuronal circuit activity. The intervention of early BDNF overexpression by doxycycline during the juvenile period can rescue the social abnormality and restore mPFC function in mice (Komori et al., 2024). Besides, microglia can indirectly facilitate synapse formation by altering the extracellular matrix through their phagocytic activity, thereby creating suitable spaces for synaptic connections. For instance, neurons in the hippocampus release IL-33 to draw microglia, prompting them to phagocytose the extracellular matrix that surrounds the neurons, thereby creating space for the formation of dendritic spines (Nguyen et al., 2020). Astrocytes and oligodendrocyte precursor cells (OPCs) also have the ability to engulf synapses. However, the drug-mediated inhibition of microglia significantly reduces the synapse engulfing capacity of these cell types (Auguste et al., 2022; Xiao et al., 2022). Thus, it is probable that a regulatory link exists between microglia and OPCs/astrocytes, which coordinates the phagocytosis of synapses, potentially through the involvement of MEGF10. Further investigation is needed to elucidate this mechanism (Lee et al., 2021; Xiao et al., 2022). Microglia also support remyelination after injury by regulating myelin sheath formation, thereby maintaining axonal health. Specifically, microglia can engulf the myelin debris produced by myelin sheath degradation. Therefore, microglial dysfunction could lead to myelin deterioration, which is a common phenomenon in aging and neurodegeneration (Safaiyan et al., 2016). However, microglia are not necessary for myelination during development and do not affect the number of oligodendrocytes or the initial formation of myelin. It has been reported that aging mice experience hypermyelination followed by demyelination, and this process could be associated with microglia (Hughes and Appel, 2020). However, the exact mechanisms underlying this process remain unclear. Previous studies have demonstrated that microglial subpopulations producing activin A, which belongs to the transforming growth factor-β (TGFβ) superfamily, play a role in regulating remyelination. When demyelination occurred, an examination of the oligodendrocyte pathway uncovered a disruption in the balance of the TGFβ-TGFβR1 axis (Miron et al., 2013). Thus, microglia may be essential for maintaining the health and integrity of the myelin sheath (McNamara et al., 2023; Miron et al., 2013). These complex physiological functions are often performed by specific microglial subsets during development. Therefore, it is necessary to investigate the physiological and functional distinctions exhibited by microglia under various conditions and to elucidate the gene regulatory networks responsible for these variations. These insights will not only deepen our understanding of the role microglia play during development but also in various other processes.

It has traditionally been believed that microglia are the only immune cells residing in the brain parenchyma under physiological conditions. Recent studies have shown that a small number of B and T cells are also present in normal brain tissue (Ito et al., 2019). The majority of these lymphocytes are CD4^+^ T cells, which rely on peripheral activation to filtrate into the brain. CD4^+^ T cells facilitate the transition of microglia from a fetal to an adult state, and the removal of these cells hinders the maturation of microglia, which in turn affects synaptic phagocytosis (Pasciuto et al., 2020). Meanwhile, brain-resident B cells promote the proliferation of OPCs, which affects the integrity of the myelin sheath (Tanabe and Yamashita, 2018). Microglia are the main immune cells in the central nervous system and have a variety of activation states. In homeostasis, microglia participate in synaptic pruning and immune surveillance. In a pathological state, microglia can be transformed into disease-associated microglia (DAM), which play a dual role (neuroprotective and neurotoxic) in neurodegenerative diseases (Paolicelli et al., 2022). Although these non-microglial immune cells are not abundant in the brain, they still have important functions. Moreover, these cells can also be detected in the brain during embryonic development; however, their origins and impacts on CNS development remain unclear. Further investigations are needed to determine why and how these immune cells persist in the CNS.

### Astrocyte

Astrocytes, the most abundant glial cell type in the central nervous system, are characterized by their distinctive stellate morphology (Escartin et al., 2021). These cells play a critical role in the development and maintenance of nervous system function, while also actively participating in a wide range of physiological and pathological processes. Primarily located within the brain parenchyma, astrocytes form close associations with neurons, blood vessels, and other glial cells, where they provide structural support and regulate neural activity and vascular dynamics. Additionally, they are essential in mediating functions at both the brain parenchyma and brain boundaries (Baldwin et al., 2024; Endo et al., 2022; Khakh and Deneen, 2019).

Astrocytes modulate the activation of the downstream transcription factor NF-κB through multiple signaling pathways during CNS inflammation. This regulation is pivotal for the progression of autoimmune diseases such as experimental autoimmune encephalomyelitis (EAE) and other CNS pathologies. Pro-inflammatory mediators, including TNF-α, IL-1β, IL-17, reactive oxygen species (ROS), and sphingolipid molecules such as sphingosine-1-phosphate (S1P) and lactosylceramide (LacCer), drive NF-κB activation via distinct mechanisms. Targeted inhibition of key molecules like S1PR1 and B4GALT6 has been shown to mitigate astrocyte hyperactivation and the associated inflammatory response. Furthermore, the aryl hydrocarbon receptor (AHR) attenuates NF-κB signaling through dietary metabolites, particularly tryptophan-derived metabolites, thereby restricting CNS inflammation. Loss of AHR function exacerbates EAE severity and upregulates the expression of pro-inflammatory cytokines and inflammatory markers, underscoring the critical role of sphingolipid signaling and AHR-mediated pathways in astrocyte regulation during CNS inflammation (Giovannoni and Quintana, 2020; Linnerbauer et al., 2020).

Mitochondrial oxidative phosphorylation (OxPhos) in astrocytes is essential for fatty acid metabolism and the maintenance of lipid homeostasis in the brain. Dysregulation of OxPhos can result in the accumulation of lipid droplets (LDs), which are associated with Alzheimer’s disease (AD) pathophysiological features, including synaptic loss, neuroinflammation, demyelination, and cognitive impairment. Excess fatty acid uptake leads to elevated acetyl-CoA levels, which activate STAT3 acetylation and drive changes in astrocyte reactivity. These alterations influence neuronal oxidative stress, microglial activation, and the synthesis of lipids necessary for myelin repair through intercellular signaling pathways (Mi et al., 2023). Findings from mouse models of AD demonstrate a strong association between lipid metabolism dysregulation, neuroinflammation, and neurodegeneration. These results highlight mitochondrial dysfunction in reactive astrogliosis is a potential central mechanism underlying neurodegenerative diseases such as AD (Habib et al., 2020).

Oligodendrocyte precursor cells (OPCs) utilize the vasculature as a scaffold for migration, with their differentiation timing tightly regulated by astrocytes through the secretion of semaphorins. Three-dimensional reconstructions have revealed that astrocytes possess a highly intricate spongiform morphology, characterized by synaptic contacts that extend from the cell body to lobules, forming contact surfaces of varying sizes with neighboring cells. Despite having relatively few internal vesicles, astrocytes exhibit a dense mitochondrial network comparable in volume to that of neuronal neurites. These findings shed new light on the multifaceted roles of reactive astrocytes in signaling, metabolic regulation, and their interactions with neurons and other astrocytes (Aten et al., 2022; Su et al., 2023). Hippocampal neuronal hyperactivity is a hallmark of early-stage AD. Amyloid-β induces astrocyte hyperactivity by TRPA1 channels, subsequently leading to neuronal hyperactivity. In the APP/PS1-21 mouse model, chronic inhibition of TRPA1 channels using HC030031 was shown to restore normal astrocytic function, prevent the retraction of perisynaptic processes, and preserve synaptic structural integrity, thereby mitigating neuronal dysfunction (Paumier et al., 2022). These findings highlight that astrocyte hyperactivity contributes to AD progression by disrupting synaptic and neuronal function. Targeted inhibition of TRPA1 channels offers a promising therapeutic strategy to slow disease progression and enhance neuroprotection. Astrocytes not only play a supporting role in the central nervous system, but also participate in neuroinflammatory regulation, synaptic plasticity, and metabolic support.

Our understanding of astrocytes remains limited, highlighting the need for future research to further explore their roles across various neurological diseases and processes. Astrocyte reactivity appears to be shaped by polarizing factors and cell–cell interactions, which are influenced by the specific characteristics of the CNS microenvironment.

### The brain border is the epicenter of immune-neural interactions

The CNS has a unique immune system. Recent observations have demonstrated the importance of brain boundaries (e.g., the meninges, choroid plexus, perivascular space, and bone marrow) in immune surveillance.

### Border-associated macrophages

**Figure 2:**
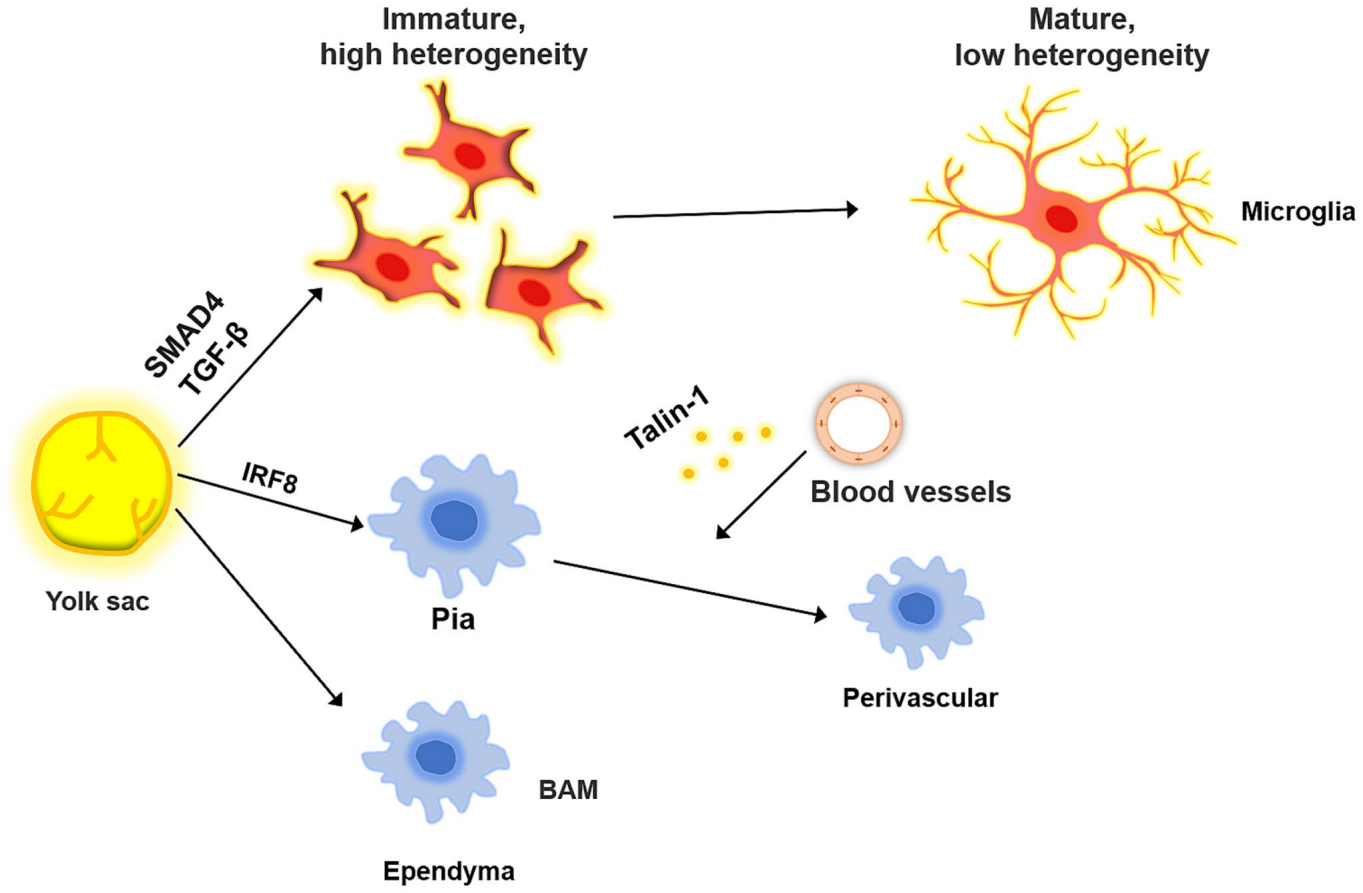
The common origin of microglia and BAM. BAM and microglia both originate from the yolk, and their differentiation is regulated by a variety of molecules. When microglia migrate to the brain parenchyma, they are in a highly heterogeneous state, which decreases as they mature. In the process, the microglia gradually acquire immune-related phenotypes. BAM migrates to the meninges and ependyma, where it influences the migration of leptomeningeal BAM to the perivascular area under the influence of blood vessels.

Bordering the central nervous system, BAMs are a diverse population of macrophages found in the leptomeninges, perivascular spaces, ependyma, and dura mater (Figure 2). Some studies suggest that BAMs, similar to microglia, are derived from the bone marrow progenitors of the yolk sac (Siret et al., 2022). The leptomeningeal and perivascular macrophages persist throughout adulthood, whereas the ependymal and dural macrophages are gradually replaced by blood-derived monocytes (Munro et al., 2022). Dural macrophages are not only derived from erythromyeloid progenitors in the yolk sac but also from monocytes in bone marrow, as the dura mater contains lymphatic and blood vessels directly connected to the bone marrow in the skull (Cugurra et al., 2021; Masuda et al., 2022). Both microglia and BAM originate from primitive macrophages produced during the first round of hematopoiesis from the yolk sac at embryonic day 7 (E7), rather than from monocytes generated during subsequent hematopoiesis round (Utz et al., 2020). Notably, the primordial macrophages differentiate into CD206^+^ macrophages at E10.5. When CD206^−^ cells became microglia, the CD206^+^ population differentiated into BAMs and ventral macrophages (Hammond et al., 2019; Utz et al., 2020). Perivascular macrophages do not directly migrate into the perivascular space. Instead, they arise from meningeal macrophages, a subpopulation that arises after birth (Masuda et al., 2019). Recent studies have highlighted the importance of transcription factors such as interferon regulatory factor 8 (IRF8), TGFβ signaling, and Talin-1 in the differentiation of microglia and their association with BAMs. For instance, conditional IRF8 knockout mice exhibited aberrant microglial development and elevated expression of the BAM-associated genes IRF7 and RUNX3 (Van Hove et al., 2019). TGFβ is an important molecule in the development of microglia and is essential for their differentiation and proliferation. TGFβ receptor depletion can alter the microglial phenotype, while TGFβ signaling is not required for BAM development or maintenance (Arnold et al., 2019). Talin-1 is important for the migration of meningeal macrophages to perivascular areas. Although Talin-1 deletion does not affect the number of microglia and meningeal macrophages, it reduces the number of perivascular macrophages (Masuda et al., 2022; Utz et al., 2020). SMAD Family Member 4 (SMAD4) influences microglial development, as evidenced by the inhibition of early microglial development upon SMAD4 knockout, without altering BAM numbers (Brioschi et al., 2023). Extracellular environmental factors serve as crucial cues for BAMs migration, a process that is highly heterogeneous with obvious tissue-specific transcriptional features, indicating a potential interaction between these components (Van Hove et al., 2019). For example, while vascular smooth muscle cells are essential for the correct distribution of perivascular macrophages, they do not contribute to the migration of microglia or meningeal macrophages (Masuda et al., 2022). Therefore, further investigation into the potential interactions and molecular signals between extracellular environmental factors and macrophages in the brain is necessary. BAMs are involved in a range of functions, including antigen engulfment and presentation, as well as monitoring the state of the CSF (Rustenhoven et al., 2021). Recent studies have demonstrated that BAMs can also regulate CSF flow dynamics through extracellular matrix (ECM) remodeling and arterial pulsation, which can further affect neural stem cells (NSCs) in the embryonic and adult stages (Drieu et al., 2022; Paridaen et al., 2013). Thus, modulating neurodevelopment through CSF is one of the pathways by which BAMs exert their functions (Silva-Vargas et al., 2016).

### Meningeal immunity and its impact on the nervous system

**Figure 3:**
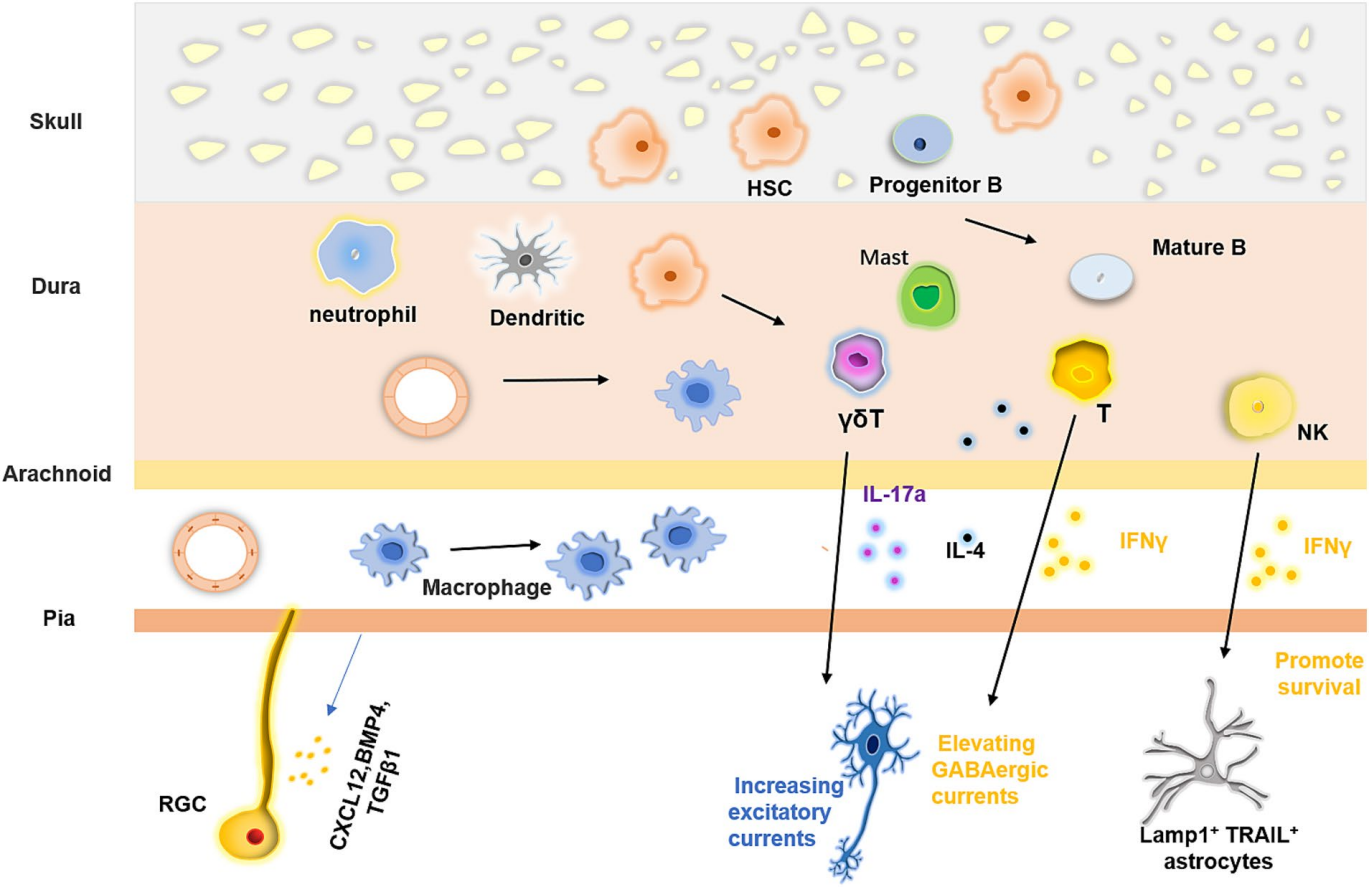
Meningeal immunity and its regulatory effect on the nervous system. Meninges possess vast and intricate communities of immune cells, which originate from diverse origins. These cells can migrate from the dural blood vessels and the skull or ascend from the proliferative and differentiative processes of stem cells within the meninges. The blood vessels situated in the pia and dura mater vary in their characteristics, the latter has fewer tight junctions that allow myeloid cells to pass through, but the former do not. Furthermore, the immune cell populace in the meninges exerts a regulatory impact on neural tissue formation by releasing various cytokines.

The brain is encased by meninges, which consist of three distinct layers: the dura, arachnoid, and pia. These membranes are situated between the skull and the brain parenchyma. The development of the meninges has been well characterized (Dasgupta and Jeong, 2019; Siegenthaler and Pleasure, 2011). The meninges originate from the mesenchymal sheaths at E9.5, which serve as precursors to the meninges, skull, and skin. These mesenchymal cells are then divided into different layers, the inner layer of which can differentiate into mature meningeal structures. At E13, the pia, arachnoid membranes, and dura can be distinguished (Siegenthaler and Pleasure, 2011). Meninges are composed of a highly heterogeneous population of cells, including vascular and lymphatic endothelial cells, smooth muscle cells, pericytes, BAMs, and different types of immune cells (Figure 3). B cells, T cells, dendritic cells, mast cells, neutrophils, and macrophages can be found in the meninges. After birth, the meningeal lymphatic vessels and structures gradually appear (Dasgupta and Jeong, 2019; Siegenthaler and Pleasure, 2011). Through techniques such as single-cell RNA sequencing and flow cytometry, a comprehensive map of immune and histological meningeal structures has been constructed.

Unlike the brain parenchyma, the meninges are capable of mounting a robust immune response. Specifically, antigens derived from the hydrocephalus can be captured by the antigen-presenting cells in the meninges and presented to T cells in the dura sinus. This process facilitates immune surveillance of the brain parenchyma (Rustenhoven et al., 2021). Moreover, the meninges also have an intact lymphatic structure. By monitoring the macromolecules present in the CSF, brain-derived metabolic waste and antigens can be removed via the lymphatic vessels (Ahn et al., 2019; Louveau et al., 2018). The dura mater exhibits varying distributions of immune cells across its distinct layers, with the highest level of immune cell diversity being observed within this structure. Additionally, the dura is equipped with both lymphatic and vascular systems (Li et al., 2022). Consequently, the dura is considered to be an important platform for immune surveillance in the CNS (Merlini et al., 2022). Meningeal immune cell populations have diverse origins. Some immune cells are derived from the blood, as the dura lacks a blood-brain barrier. In addition, blood vessels in the dura express high levels of leukocyte chemokines and low levels of tight junctions, which contribute to the transport of immune cells, especially under inflammatory conditions. The trafficking of immune cells via dural sinuses is an important route for immune surveillance (Rustenhoven et al., 2021). Other immune cells originate from the bone marrow. The cranial bone marrow is anatomically connected to the dura mater through numerous channels, which allow for the continuous supply of myeloid cells to the meninges (Herisson et al., 2018). Whether under inflammatory conditions or in a state of homeostasis, the meninges actively produce a range of chemokines that attract neutrophils, monocytes, B cells, and macrophages to the meninges (Brioschi et al., 2021; Cugurra et al., 2021). The meninges harbor various subsets of B cells, including progenitor B cells, pre-B cells, and immature and mature B cells. These cells, which originate from the bone marrow of the skull, migrate to the meninges, where they mature. Unlikely peripheral B cells, CNS-derived B cells are present at different stages of development and require further maturation to function. B cells in the meninges that recognize CNS self-antigens undergo negative selection, which eliminates autoreactive cells and contributes to the establishment of a non-autoreactive CNS immune microenvironment (Brioschi et al., 2021; Wang et al., 2021). Furthermore, some immune cells arise from progenitor cell proliferation. For example, BAMs can persist and proliferate for a long time after migrating to the pia in early development. In addition, hematopoietic stem cells (HSCs) are present in the meninges after birth and express hematopoietic niche factors locally. Meningeal HSCs retain their local stability and can be activated to replenish immune cell populations when the existing immune cells are reduced (Niu et al., 2022).

Recent studies have identified a type of lymphoid membrane in the subarachnoid space, called the subarachnoid lymphatic-like membrane (SLYM), which lies between the arachnoid and leptomeningeal spaces. SLYM consists of a single layer of cells interspersed with loose collagen fibers. It divides the subarachnoid space into two compartments (an upper surface and a lower deep chamber), functioning as a barrier that impedes the entry of molecules larger than 3 kD into either chamber. SLYM contains a variety of immune cells, including leukocyte cells, macrophages, and dendritic cells, suggesting that it serves as a critical microenvironment for immune cells to function (Herisson et al., 2018).

The meninges play a vital role in brain development. During early brain development, they release cytokines such as CXCL12, BMP4, BMP7, and TGFβ1, which modulate the proliferation and differentiation of radial glial cells and govern the migration and positioning of neurons. Meningeal fibroblasts also regulate the production of cortical neurons by secreting retinoic acid (Borrell and Marín, 2006; Radakovits et al., 2009). In addition to mediating neural development through immune factors, RGCs can also directly interact with the pia via their fibers. These fibers provide scaffolding for neurons, allowing them to migrate within the cortex. Recent studies have shown that, during early development, neural stem cells can migrate to the meninges and produce neurons that integrate into the neural circuitry. Direct and complex interactions between NSCs and various meningeal cells occur during this process although the precise mechanisms remain unclear (Decimo et al., 2021). While the roles of many meningeal immune cells in neural development are not yet fully understood, they are known to produce a large array of cytokines that influence neuronal physiology and function. For example, IL-17a, produced by γδ T cells in the postnatal dura, binds to its receptor on cortical glutamatergic neurons, increasing the frequency of excitatory postsynaptic currents. This mechanism helps maintain anxiety-like status and facilitates spatial learning (Ribeiro et al., 2019). Furthermore, IL-17 promotes brain-derived neurotropic factor production by glial cells, and mice lacking γδ T cells or IL-17 exhibit short-term memory impairment (Alves de Lima et al., 2020). A large number of meningeal T cells also express IFNγ. In response to meningeal T cell-derived IFNγ, inhibitory neurons enhance GABAergic currents in projection neurons, ultimately modulating social behavior (Filiano et al., 2016). IL-4 produced by CD4^+^ T cells plays a role in regulating learning and memory (Derecki et al., 2010; Herz et al., 2021). Moreover, meningeal natural killer cells produce IFNγ to sustain the survival of Lamp1^+^ TRAIL^+^ anti-inflammatory astrocytes, which are associated with inflammation under pathological conditions (Sanmarco et al., 2021).

Despite significant advances, few studies have explored the relationship between immunity and meningeal development. Thus, many questions surrounding meningeal development remain unresolved. Recent findings indicate that the three layers at the base of the meninges are primarily composed of fibroblasts. These fibroblasts, which make up the dura, arachnoid, and leptomeningeal membranes, can extend across the entire forebrain by as early as E14. Notably, this period coincides with the peak of nerve trunk differentiation into neurons, suggesting that there may be a relationship between these processes (DeSisto et al., 2020). Since astrocytes emerge later in development, the boundary between the meninges and the parenchyma (the leptomeninges and the glia limit are formed by the astrocytes) is not yet established at this period. However, some myeloid cells, such as macrophages, are present in the meninges at this time point. Therefore, the injection of LPS at E9.5 may influence microglial development during the embryonic period. Whether meningeal macrophages are affected and whether neurodevelopment is indirectly impacted through these cells remains to be determined (Hayes et al., 2022). Although much research has been conducted on the immune niche within the meninges that spans the period from birth through adulthood, the composition and distribution of immune cells in the meninges during early embryonic development, and their contribution to brain development, remain poorly understood. Therefore, the relationship between early meningeal immune function and brain development represents a new research direction that is worth pursuing (Masuda et al., 2022).

### Immunity and neurodevelopmental diseases

**Figure 4:**
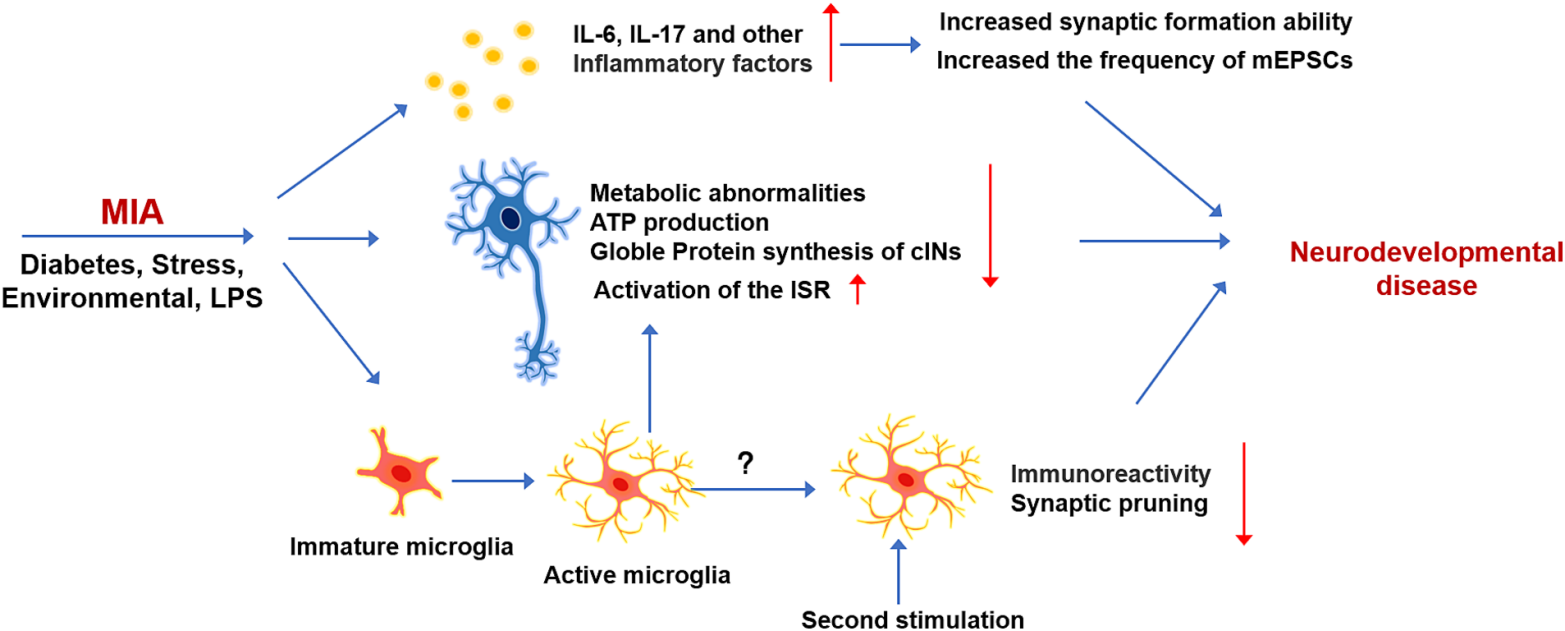
The effect and mechanism of MIA on neurodevelopment. Early immune stimulation, which is induced by LPS or other environmental factors, can exert long-term effects on the brain development of offspring. MIA can induce the production of various Inflammatory cytokines, which can affect the development of neurons, metabolism, and synaptic excitability. Immature microglia respond to immune stimulation in advance, which can affect the normal development of microglia and have long-term effects on microglia responsiveness.

The interaction between the immune and nervous systems implies that alterations in immune system function are closely linked to nervous system disorders. Early epidemiological studies investigations into the consequences of prenatal infection exposure on neurological disorders are based on data gathered from the 1957 influenza pandemic. This study found a significant increase in the prevalence of neurodevelopmental disorders, such as autism spectrum disorder (ASD) and schizophrenia, rising from less than 1% to approximately 10% following infection (Patterson, 2009). Since this pioneering study, increasing evidence has shown that maternal infections during early fetal development are associated with various cognitive disorders. A specific mechanism termed maternal immune activation (MIA) has been identified as a potential driver of neurodevelopmental disorders and mental health issues in offspring (Figure 4). The adverse effects of MIA may manifest from the embryonic stage through adulthood (Estes and McAllister, 2016). Experimental models of MIA have been developed commonly through the injection of bacterial polyribonucleotide (poly I:C) or lipopolysaccharide (LPS) into pregnant animals (Bao et al., 2022). These agents activate Toll-like receptors (TLRs), triggering a maternal inflammatory response that leads to behavioral and cognitive impairments in the offspring (Talukdar et al., 2021). Recent research has also revealed that various factors contribute to MIA, including maternal diabetes, stress, and environmental risk factors (Marques et al., 2015; Patel et al., 2021). These different factors can induce abnormal maternal immunity and autism-like symptoms in the offspring. However, the complexity of the human nervous system and its sensitivity to environmental influences have led some researchers to propose that MIA may interact with genetic mutations and environmental factors. This interaction could further predispose individuals to disease-related symptoms later in life (Knuesel et al., 2014; Nielsen et al., 2014).

The mechanisms by which immune dysregulation affects fetal development remain incompletely understood. However, findings from MIA model studies suggest that alterations in cytokine levels play a critical role. Notably, two key proinflammatory cytokines, IL-6 and IL-17, are capable of crossing the placental barrier and binding to receptors on fetal brain cells, potentially initiating neurodevelopmental disorders (Choi et al., 2016; Qing et al., 2020). Elevated IL-6 levels have been consistently observed in the serum and CSF of adults diagnosed with neurodevelopmental disorders such as autism and schizophrenia. This observation implicates chronic IL-6 elevation as a significant contributor to the development of neurological abnormalities. IL-6 prenatal administration enhances glutamatergic synapse formation and functional connectivity in the adult hippocampus. Single-cell sequencing has revealed that IL-6 may exert a direct effect on excitatory neurons, selectively promoting synaptic formation. Specifically, IL-6 incubation significantly increases the frequency of miniature excitatory postsynaptic currents (mEPSCs), without affecting miniature inhibitory postsynaptic currents (mIPSCs). The process is mediated by a signaling pathway that involves the activation of STAT3-RGS4 (Mirabella et al., 2021). Additionally, cytokines such as IL-1β, IFN-γ, TNF-α, and IFNs can trigger MIA by being secreted in both maternal and fetal mouse tissues. While these immune factors have important regulatory roles in neurodevelopment under physiological conditions, their dysregulation may contribute to the onset of MIA or other neurodevelopmental diseases. Consequently, it is essential to systematically evaluate the modulatory roles of these cytokines across diverse conditions to better understand their impact on neurodevelopment.

Maternal infection during early stages of development profoundly impacts offspring, potentially inducing permanent cellular changes at the molecular level. MIA can directly affect the development and physiological function of neurons. During the initial stages of inflammation, the activation of microglia and the secretion of inflammatory factors can affect neurodevelopment. However, in MIA animal models, the inflammation is typically induced in the early stages of development, before microglia have fully matured. Consequently, early immune system activation may impair microglial development and maturation, thereby affecting their normal physiological functions. For instance, administering inflammatory factors to pregnant mice at E9.5 resulted in a reduced microglial response to inflammation in adult offspring (Hayes et al., 2022). Early developmental infections can compromise the ability of microglia to prune synapses. Studies demonstrate that microglia during embryonic immunity exhibit a diminished capacity to engulf thalamocortical glutamatergic synapses compared to microglia later in life. This phenomenon is not a contribution to a general reduction in CD68 expression levels but rather to a change in the CD68 distribution pattern, indicating that different microglial subsets may exhibit varying synapse pruning capacities. Thus, early immune stimulation can affect microglial heterogeneity (Loayza et al., 2023). In addition to these effects, microglia can regulate the function of cortical interneurons, potentially causing metabolic abnormalities. For instance, when developmental cortical interneurons are co-cultured with microglia, they exhibit increased expression of connective tissue growth factor (CTGF) and thrombospondin 19 (THBS19). These factors are known to inhibit the metabolism of cancer cells and adipose tissue, respectively. The upregulation of these factors impairs the cellular respiration capacity of interneurons, reducing their ability to generate ATP. Notably, the glutamatergic neurons are unaffected by co-culture with microglia, suggesting that specific microglial populations may selectively target certain neuronal subtypes (Park et al., 2020). Single-cell sequencing of fetuses from mothers with MIA reveals that immune stimulation alters protein synthesis in the brain and activates the integrated stress response (Qing et al., 2020). ISR, which can be activated under both physiological and pathological conditions, reduces the rate of protein synthesis as a means of diverting resources toward cell survival. The phosphorylation of eIF2α, a key hub for ISR activation, is significantly increased following MIA-mediated ISR activation. The administration of IL-7a-blocking antibody completely suppressed MIA-induced eIF2α phosphorylation in the male fetal cortex, restoring protein synthesis to normal levels (Kalish et al., 2021).

Rodent studies employing the two-hit model can provide unique insights into the pathogenesis of neurodevelopmental disorders induced by immune stimulation. In the two-hit MIA model, repeated immune stimulation is used to sequentially induce MIA and postnatal immune activation. The exposure to inflammation during early developmental stages and postnatal period leads to the manifestation of behavioral abnormalities in the offspring. The upregulation of various inflammatory cytokines in the fetal brain after the first round of immune stimulation undoubtedly disrupted the development of various types of cells in the nervous system (Ben-Yehuda et al., 2020). For instance, early activation of immature microglia alters their subsequent response to immune triggers during the postnatal period. The alternation of microglial state may increase the organism’s susceptibility to future immune challenges, potentially contributing to the onset of neurodevelopmental disorders. Despite these findings, the precise long-term effects of early-stage immune activation on microglial function remain incompletely understood. Further research is required to fully elucidate how such early immune disturbances impact microglial behavior and its role in neurodevelopmental pathologies (Ben-Yehuda et al., 2020; Hayes et al., 2022).

### ASD

ASD is a prevalent neurodevelopmental disorder characterized by ineffective communication, restricted interests, repetitive behaviors, and impaired social interactions (Lord et al., 2018). The etiology of ASD is complex, involving complex interactions between environmental and genetic factors that result in developmental and functional abnormalities of the nervous system. Inflammation and immune system dysregulations are commonly observed clinical features of ASD, with effects extending beyond the CNS to other organ systems (Masini et al., 2020). One of the key aspects of immune dysfunction in ASD is the dysregulation of the immune response within the CNS, which includes elevated concentrations of proinflammatory cytokines and the state of microglia and astrocytes. Notably, neuroinflammation has been consistently detected in postmortem brain tissue from individuals with ASD, spanning a wide age range (4–45 years). This includes significant microglial response and increased levels of inflammatory cytokines such as IL-6, IL-1β, TNF-α, and IFN-γ. Although the mechanisms underlying the contribution of these immune factors to autism development remain incompletely understood, emerging evidence suggests that abnormal immune function leads to developmental and physiological disorders of neurons and neuroglia in the brain (Becher et al., 2017). For instance, TNF-α, produced by microglia, astrocytes, and other cells in response to inflammation, significantly impacts the physiological condition of the CNS. Beyond its role in orchestrating inflammation, TNF-α regulates processes such as neuronal cell proliferation, differentiation, apoptosis, and synaptic pruning (Linnerbauer et al., 2020; Liu et al., 2017). Other proinflammatory cytokines, including IL-6, IL-1β, and IFN-γ, exert diverse effects on the neuronal survival, proliferation, differentiation, synapse formation, and migration (Ng et al., 2018; Patel et al., 2019). Under physiological conditions, astrocytes and microglia are essential for maintaining CNS homeostasis. However, in individuals with ASD, these glial cells become primary mediators of the inflammatory response in the CNS (Kwon and Koh, 2020). Extensive research has shown that these changes in astrocyte function contribute to the disruption of CNS homeostasis, leading to persistent reactive of microglia and astrocytes to chronic neuroinflammation and neuronal damage (Araki et al., 2021; Pekny and Nilsson, 2005). The neuroinflammatory state and neuroglial involvement in ASD are characterized by phenotypic changes, suggesting a central role for glial cells in the pathology of the disorder. Astrocyte responsiveness may be a result of the local neuronal dysfunction caused by ASD, which could exacerbate pre-existing synaptic and axonal abnormalities (Allen and Eroglu, 2017). Alternatively, glial cells, including astrocytes, may respond to environmental cues, such as MIA (Esshili et al., 2020). While ASD is primarily associated with impairments in brain function, the evidence of widespread immune dysfunction indicates that other systems, including immune polymorphisms influencing immune cell activity, microglial and astrocytic homeostatic, and proinflammatory cytokine production, are also disrupted. Despite these insights, the precise relationship between ASD and immune system dysfunction remains unclear and warrants further investigation in future studies.

### Schizophrenia

Schizophrenia is a severe and heterogeneous mental disorder characterized by symptoms such as thought disturbances, avolition, social withdrawal, and cognitive impairments (Winship et al., 2019). While the precise etiology of schizophrenia remains elusive, growing evidence suggests that the disorder is significantly influenced by interactions between the immune and nervous systems, akin to other neurodevelopmental disorders (Müller, 2018). Alterations in cytokines profile have been observed in the CSF of individuals with schizophrenia (Upthegrove and Khandaker, 2020). As in ASD, patients with schizophrenia exhibit elevated levels of proinflammatory cytokines, such as IL-1β, IL-6, and TNF-α, alongside reduced levels of anti-inflammatory cytokines, including IL-10, IL-1β, and IL-8, when compared to healthy individuals (Gallego et al., 2018). In addition to cytokine dysregulation, recent studies have implicated the complement system in the development of schizophrenia. Complement pathway activity is significantly elevated in individuals with the disorder, with the overexpression of complement component 4A (C4A) being particularly noteworthy (Yilmaz et al., 2021). These findings underscore the potential role of immune dysregulation, particularly involving cytokines and the complement system, in the pathogenesis of schizophrenia. Further exploration of these immune-related mechanisms may yield valuable insights into the development and treatment of this complex neurodevelopmental disorder.

## Discussion

Neuroimmunology is an emerging interdisciplinary field that has made many advances in the past few decades, revealing the important role of the immune system in nervous system development and functional regulation (Lukens and Williams, 2022). However, despite the remarkable achievements of neuroimmunology research, there are still some limitations and challenges. Some questions still need to be explored. The interaction of neuroimmunity is very complex, involving a variety of immune cells as well as different cell types within the nervous system. Immune responses can be a double-edged sword, both protective and potentially leading to pathological nerve damage (e.g., chronic neuroinflammation) (Lecca et al., 2022). The delicate regulation of this balance makes the study of neuroimmune mechanisms more difficult. Immunotherapies for neurodevelopmental disorders are still in the exploratory phase. In addition, the neuroimmune response has strong temporal and spatial dynamic characteristics. The activation of immune cells at different stages of neurological diseases may show different phenotypes and functions, and a more refined spatio-temporal dimension is needed to illustrate how the immune system affects the health and disease of the nervous system. With advances in technology and a better understanding of neuroimmune interactions, more effective therapeutic strategies to improve clinical outcomes for neuroimmune-related diseases will be developed in the future (Dyatlova et al., 2022; Kolliker-Frers et al., 2021).

The immune system of the brain can be divided into the brain parenchyma and brain border. Microglia bridge these two subsystems by interacting with various nerve and glial cells, ultimately regulating CNS homeostasis. During development, microglia are highly heterogeneous and can be divided into different groups that perform specific physiological functions (Tan et al., 2020). Although both the microglia and BAMs originate from the yolk sac, little is known about their differentiation processes. BAMs comprise macrophages located in the meninges, perivascular and ependymal regions of the brain (Stifter and Greter, 2022). How these macrophages migrate to specific niches and what functions they perform merits further investigation. As an important immune barrier in the outer layer of the brain, the meninges have a unique immune environment and can monitor the physiological activities of the brain. Of note, no astrocyte barrier exists between meninges and brain in the early stages of development, indicating that cells can enter the meninges from the brain parenchyma at that period. Immunostimulation can exert long-term effects on neurons, microglia, and other cells of the brain parenchyma. However, how immunostimulation affects the complex immune environment of the meninges is currently unclear. Thus, the long-term effects of immunostimulation on the nervous system need to be further explored (Zhang et al., 2020). Some progress has been made in the diagnosis and treatment of neurodevelopmental diseases. For instance, techniques such as genetic testing, neuroimaging, and electrophysiological assessments have been employed to obtain the early diagnosis of neurodevelopmental disorders in children. Multi-omics research and increased cooperation between multi-disciplinary clinical teams have also helped improve the early detection and timely management of neurodevelopmental disorders. However, there are still no effective measures for controlling the continuous development of clinical symptoms and limiting brain damage (Sacks et al., 2018; Sharma et al., 2018; Shen et al., 2018). Therefore, it will be crucial to clarify the pathogenesis of neurodevelopmental disorders to identify accurate diagnostic/prognostic biomarkers and develop effective treatment strategies.

## Conclusion

This review highlighted the findings and discovery about immune factors and cells in brain development and related disorders. There is a high degree of connection between the immune system and the nervous system. Various immune cells and the immune factors are extensively involved in the growth, differentiation, migration, synapse formation, and maturation of the nervous system. Therefore, the changes of immune system would directly affect nervous system function. The development of neurodevelopmental disorders is a multifaceted process, in which immune factors undoubtedly contribute significantly. The response of microglia, as well as abnormal changes in inflammatory factors in the CNS, profoundly affect the behavior and function of the nervous system. Future research may shed light on how the immune system functions at all stages of neurodevelopment and provide new ideas and strategies for the treatment of neurodevelopmental diseases, mental disorders, and neurodegenerative diseases.
